# Supplementary material for: The BACE‐1 inhibitor CNP520 for prevention trials in Alzheimer's disease
Source: EMBO Mol Med. 2018 Sep 17;10(11):e9316. doi: 10.15252/emmm.201809316 (PMC6220303; doi:10.15252/emmm.201809316)
Supplement: Supplementary file 1 — Appendix [file EMMM-10-e9316-s001.pdf]

The BACE-1 inhibitor CNP520 has a pharmacological and safety profile suitable for prevention trials in Alzheimer’s disease

Appendix

Appendix Materials and Methods

Appendix Figure S1. Initial electron-density for CNP520 bound to the BACE-1 extracellular domain

Appendix Figure S2. Effects of long-term CNP520 treatment on soluble APP metabolites in APP23 mice

Appendix Figure S3. Kinetic analysis of the disappearance of APP metabolites in CSF

Appendix Figure S4. Effects of 3-month CNP520 treatment on APP-related CSF biomarkers

Appendix Table S1. X-ray data collection and refinement statistics for the BACE-1 CNP520 complex

Appendix Table S2. Binding of CBNP520 to and its functional affinities for different targets relative to the free plasma concentration at a clinical dose of 85mg/kg/day

Appendix Table S3. Overview of completed clinical studies with CNP520

Appendix Table S4. Study synopsis of completed clinical studies with CNP520

Appendix Table S5: Incidence of AEs by primary system organ class (SOC) after single dose in healthy participants ≥ 60 years

Appendix Table S6: Incidence of AEs by primary system organ class (SOC) after single dose in healthy participants ≥ 60 years, arranged in descending order of total frequency

Appendix Table S7. Incidence of AEs by primary system organ class (SOC) after multiple, once-daily dosing over two weeks in healthy participants ≥ 60 years

Appendix Table S8. Incidence of adverse events after 3 months daily dosing in healthy participants ≥ 60 years

Appendix Table S9. Plasma PK parameters following single dose administration of CNP520

Appendix Table S10. CNP520 Plasma PK parameters following multiple dose administration to healthy participants ≥ 60 years for 14 days and for 3 months

Appendix Table S11. CSF PK parameters in healthy participants ≥ 60 years

Appendix Materials and Methods

Animal genotyping

Genotyping was performed using an APP-specific Taqman assay on genomic DNA that had been extracted from lysed tissue samples. Tail tip biopsies (2 mm in diameter) were lysed in 400 µL of proteinase-K solution (93 µg/ml in 100 mM NaCl, 10 mM EDTA, 0.2% (w/v) SDS, 50 mM Tris/HCl pH 8.0) over night at 56°C. The enzyme was heat inactivated at 85°C for 10 min and samples were centrifuged at 4000 rpm for 10 min in a 5810 R centrifuge (Eppendorf). The supernatant was diluted 1:10 in water and directly used as template for qPCR analysis.

The APP transgene was detected by Taqman using primers 176-APP fw (5'-ATCACCGCTCTGCAGGCT-3'), 177-APP rev (5'-CTTCTGTTCTGCGCGGACAT-3') and the probe 178-APP-tqmn-pb (5'-FAM-CTCCTCGGCCTCGTCACGTGTTCAAT- TAMRA-3'). BACE-1 knockout mice were identified by multiplex Taqman using primers neo-F(5'-TGGATTGCACGCAGGTTCT-3'), neo-R (5'-GTGCCCAGTCATAGCCGAAT-3'), neo-Taq (5'-FAM-CGGCCGCTTGGGTGGAGAGG-BHQ1-3'), endo-F (5'-CGGGACCACCTCCCAAA-3'), endo-R (5'-CCCCATAATGGCATCCCGAA-3') and endo-Taq (5'-YY-CTTCATCGGAGCACACCAGGCAGA-BHQ1-3'). FAM: Fluorescin, YY: Yakima Yellow, BHQ1: Blackhole Quencher 1.

Taqman analysis was performed on a "StepOnePlus" qPCR system (Applied Biosystems) using 96-well plates. The PCR program consisted of an initial step of 50°C for 10 min, followed by the initial heat denaturation at 95°C for 10 min, followed by 40 cycles of 15 sec at 95°C and 1 min at 60°C. After completion of the run, the results were analyzed using StepOne software V2.3. BACE-2 KO mice were identified by standard PCR (Taq polymerase) and agarose gel analysis (KO: 400 bp and WT 1010 bp band size). The PCR program consists of an initial step of 95°C for

10 min, followed by 30 cycles of 1 min at 95°C, 1 min at 53°C and 1 min 72°C with a final step of 10 min at 72°C. Primers used: BACE-2-KO F:5`- GCTATAGAGACCAAAGCCCACAAA TCT-3`, BACE-2-KO R: 5`-GCCCGAATAACAAGAGCATCAC-3`.

X-ray analysis of the BACE-1 complex with CNP520

The catalytic domain of human BACE-1 (residues 46–447 of Uniprot entry P56817) was expressed in *Escherichia coli* and refolded as described (Hanessian et al, 2005). The structure of the complex with CNP520 was obtained by soaking an orthorhombic crystal of unliganded BACE-1, grown at 19ºC from 15% PEG 1,500 in water by the method of vapor diffusion in hanging drop using 12.7 mg/ml BACE-1 in 10 mM Tris-HCl pH 7.4, 25 mM NaCl. The soaking buffer was 30.0% PEG 1,500, 50 mM sodium citrate pH 5.5, with 1.0 mM CNP520 and 1.8% DMSO. The crystal was directly flash frozen into liquid nitrogen and diffraction data were collected at the Swiss Light Source (Paul Scherrer Institut, Villigen, Switzerland) beamline X10SA, with a Pilatus detector. Diffraction data were processed with XDS. The structure was determined by difference Fourier using autoBUSTER (Global Phasing Ltd, Cambridge, UK) and refined by multiple cycles of electron-density map inspection and model rebuilding in Coot (Emsley et al, 2010), followed by automated refinement with autoBUSTER. See Fig. S2 for initial difference density of CNP520 and Fig. S8 for X-ray data collection and refinement statistics.

Magnetic Resonance Imaging (MRI)

Three-dimensional T2*-weighted images covering the whole brain were acquired at 7T using a Biospec 70/30 spectrometer (Bruker Medical Systems, Ettlingen, Germany) equipped with an actively shielded gradient system. The operational software of the scanner was Paravision PV5.1 (Bruker). Images were obtained using a 3D gradient-echo sequence with the following imaging parameters: repetition time 24.3 ms, echo time 10 ms, matrix 256 × 128 × 192, field-of-

view 1.5 × 1.5 × 2.4 cm^3. Total acquisition time for an image having a voxel size of 59 × 118 × 125 mm^3 was 10 minutes. Shimming (homogenization of the magnetic field) was performed for every animal and imaging session before acquiring a 3D image. During MRI acquisitions, mice were anaesthetized with isoflurane (Abbott, Baar, Switzerland) administered via a face mask and placed in a cradle made of Plexiglas. The head was fixed by using a tooth holder. Body temperature was maintained at 37 ± 1ºC *via* warming blankets or integrated water hoses in animal beds. Respiration and body temperature were monitored throughout the acquisition. The duration of an imaging session was about 15 minutes, including positioning of the mouse.

Sites in the cortex and thalamus presenting signal attenuation with a minimum diameter of 150 mm were analyzed (counts, volumes) throughout the whole brain. To ensure that the same site was not counted multiple times, its presence was carefully controlled over several consecutive slices from the 3D data set. The lesion volume was determined using specialized software (ImgTool), developed in house. A detailed description of the software can be found in (Babin et al, 2012; Egger et al, 2013). The software has been validated and is in routine use since 2001. For each slice of the 3D data set, the areas of the signal attenuation sites within an external border were determined by applying an image segmentation algorithm. The total volume of the lesions was obtained by adding the areas assessed on the individual slices and multiplying by the slice thickness (125 mm).

Statistical analyses of lesion volumes comprised analysis of variance (ANOVA) with random effects (SYSTAT 12, Systat Software, Inc, San Jose, CA, USA) to take into account the longitudinal structure of the data. Manne Whitney analyses (SYSTAT 12) were performed on lesion numbers; $p < 0.05$ was considered statistically significant.

Immunofluorescence

Amyloid plaques were stained using the Novartis-internal rabbit anti-Aβ antibody NT12, which recognizes all forms of Aβ (Neumann et al, 2015). Activated astrocytes were detected using a commercial rabbit anti-GFAP (Z0334, Dako Schweiz GmbH, Baar, Switzerland, dilution 1:2000 in antibody diluent). Microglia cells were detected using a rabbit anti-Iba1 antibody (019-19741, Wako Chemicals GmbH, Neuss, Germany, dilution 1:200 in antibody diluent). All stainings were performed using the fully automated instrument Ventana Discovery® Ultra (Roche Diagnostics Schweiz AG, Rotkreuz, Switzerland). All chemicals were provided by Roche Diagnostic. Paraffin-embedded brain tissue sections of 3 µm were freshly cut and collected on SuperFrost+ slides. The tissues sections were de-paraffinized and rehydrated under solvent-free conditions (EZprep solution) followed by antigen retrieval (demasking) performed by heat retrieval cycles for 32 min in EDTA-based buffer (CC1 solution). Subsequently, slides were blocked for 4 min using the DISC Inhibitor RUO (reference 07017944001). The primary antibody anti-NT12 diluted at 1:20 000 in antibody diluent was manually added to tissue sections and incubated for 1h at room temperature. A short post-fixation (glutaraldehyde at 0.05%) was done before applying the multimer UltraMap-anti Rabbit HRP ready to use antibody (reference 05269717001) for 16 min.

Detection was performed using the DISCOVERY FITC® following the manufacturer's recommendations. Slides were then heat denaturated at 92°C for 20 min before a manual application of the second primary antibody (anti-GFAP diluted at 1:2000, anti-Iba1 diluted at 1:200) and incubated for 1h at room temperature. Sections were then incubated for 20 min at room temperature with UltraMap-anti-Rabbit HRP antibody, and the DISCOVERY Rhodamine kit (reference 07259883001) was used to detect GFAP.

The slides were washed and mounted using Prolong® Gold antifade reagent (P36931, ThermoFisher, Switzerland) and further scanned with the Hamamatsu slide scanner instrument

(NanoZoomer 2.0 HT, scanning software NDP-Scan Version 2.5, Hamamatsu Photonics France, Swiss Office, Solothurn, Switzerland) at the 40x objective. The scanning settings were as follow: the exposure time with the DAPI filter was set at 57 ms, as was the FITC filter. The exposure time for the TRITC filter (detection of Rhodamine) was set at 14.2 ms.

Image Analysis

For the quantitative plaque and blood vessel-associated Aβ evaluation based on image analysis, a proprietary image analysis platform (ASTORIA, Automated Stored Image Analysis) was developed based on MS Visual Studio 2010 and many functions from Matrox MIL V9 libraries (Matrox Inc, Quebec, Canada).

For the Aβ plaque, neuroinflammation and blood vessel analyses, the following sequence of steps was performed. (1) Slides were scanned with Hamamatsu Nanozoomer at 40x magnification. For each fluorescence labelling (DAPI, FITC and TRITC), a separate image was created. (2) ROIs (regions of interest) were manually outlined for define cortex in brain sections for Aβ plaque assessment on the green FITC channel image and the resulting outline was also used for the other two channel images (copy resulting xml files). (3) The ImageScope (V12.1.0.5029, Aperio Inc., USA) plug-in that was developed in house was run to create and export *.tif image tiles (at 10x magnification) for each of the 3 fluorescence channels. (4) Image batch processing was then performed as follows: (a) the combined true color image (DAPI, FITC, TRITC) for each section was obtained through accessing each individual fluorescence channel image; (b) valid samples (within outlined ROI) were segmented from black unstained background; and (c) adaptive thresholding technique was applied to segment the objects in the green channel image (FITC-labeled Aβ plaques).

For GFAP and Iba1 staining, the process was as follows: (a) to allow subsequent analysis, objects that were touching were separated by elimination of debris with signal in the green (FITC) channel; (b) TRITC-labeled objects in the red channel (specific for GFAP or Iba1 staining indicating astrocytes or microglia) were segmented through morphological tophat transformation and thresholding; (c) feature-based object classification was performed.

For GFAP+NT12 and Iba1+NT12 staining the process was as follows: (a) Identification of 4 object categories for plaques — unspecific debris (too faint, too small objects) to be excluded, small plaques (40 ... 1000 pixels), medium plaques (1000 ... 6500 pixels), large plaques (> 6500 pixels). (b) Computation of the following morphometric and densitometric features for valid plaques: Number of plaques; “specific optical density” which has been described to reflect the amount of protein (antigen) concentration, based on measuring and using the staining intensity of an appropriate antibody in a non-linear way (Rahier et al, 1989; Ruifrok & Johnston, 2001); assessment of “plaque-associated GFAP or Iba1” based on the ratio of TRITC+ signal *vs* plaque area; “proximal GFAP or Iba1” based on ratio of TRITC+ signal within dilation ring around plaque.

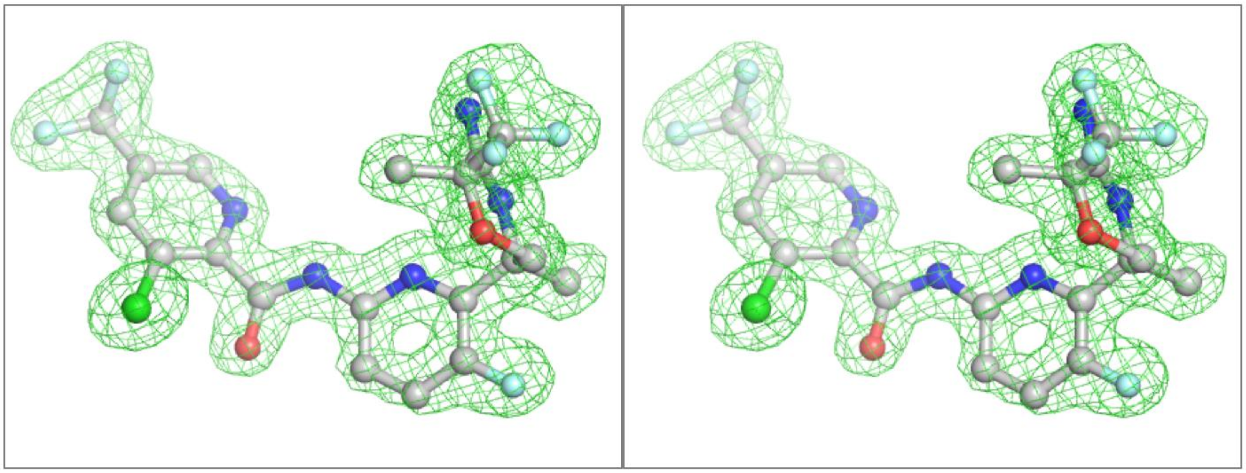

Appendix Figure S1. Initial electron-density map of CNP520 bound to BACE-1 extracellular domain. Stereo view showing the initial (Fo-Fc, ϕcalc) electron density map, 5.0 σ contour. PDB code: 6EQM

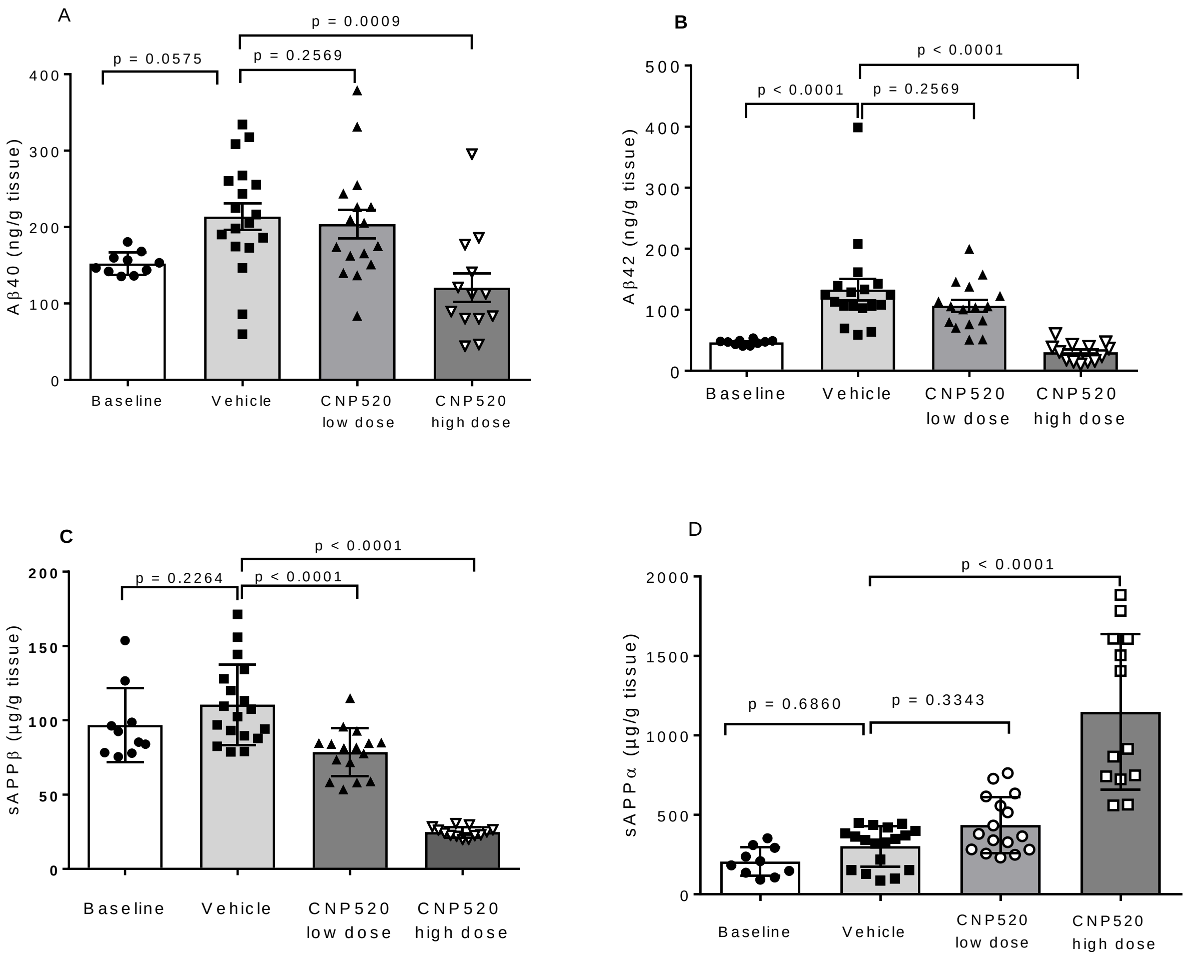

Appendix Figure S2. Effects of long-term CNP520 treatment on soluble APP metabolites in APP23 mice. Mice were dosed with CNP520 in food pellets. Low dose: 0.03 g CNP520/kg food, exposure corresponds to a daily oral dose of 4 mg/kg. High dose: 0.3 g/kg food, exposure corresponds to a daily oral dose of 40 mg/kg. At the end of the study, half-brains were homogenized, and extracted with 1% Triton X-100. After appropriate dilution, extracts were analyzed using MesoScale Diagnostics kits, using 6E10 anti-Aβ antibody kit for Aβ40 and Aβ42, and for sAPPα and sAPPβ using the respective kits and manufacturer's protocols. (**A**) Soluble Aβ40. (**B**) Soluble Aβ42. (**C**) soluble sAPPβ (Swedish). (**D**) soluble sAPPα. Data were analyzed with one-way ANOVA and Dunnett's multiple comparison test.

A B

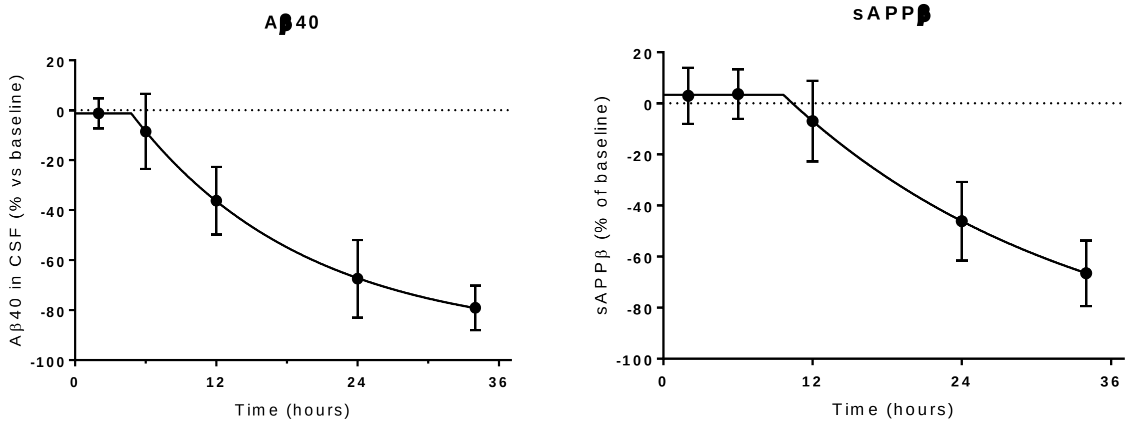

Appendix Figure S3. Kinetic analysis of the disappearance of APP metabolites in human CSF after a 750 mg CNP520 dose. Time-metabolite data pairs were fitted to a model of plateau-phase followed by single exponential decay (GraphPad Prism) and yielded the first-order rate constant k. (**A**) Aβ40, k = 0.068 ± 0.002 h^{-1} (**B**) sAPPβ, k = 0.043 ± 0.004 h^{-1}. Shown is average ± SD, n=6 at each time point.

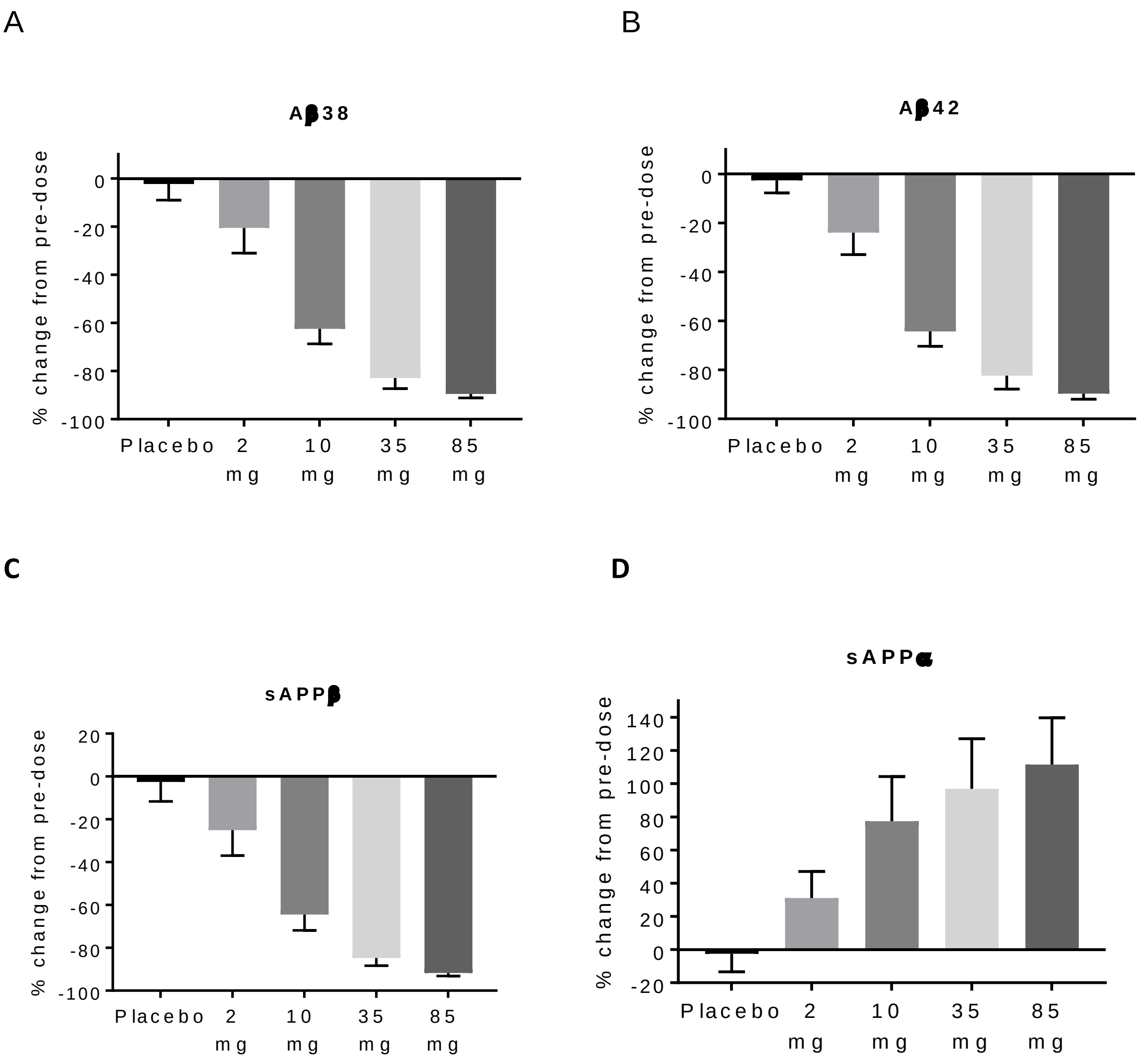

Appendix Figure S4. Effects of 3-month CNP520 treatment on APP-related CSF biomarkers. (**A**) Aβ38, (**B**) Aβ42 (**C**) sAPPβ (**D**) sAPPα. CSF was collected by lumbar puncture 24 hours after last dose from 20–23 participants/dose group. Biomarkers were determined using Mesoscale Discovery Inc. assay kits according to manufacturer's protocol. Shown are means ± SD.

Appendix Table S1. X-ray data collection and refinement statistics

	BACE-1 complex with CNP520
**Data collection**	
Space group	$P2_12_12_1$
Cell dimensions	
a, *b*, *c* (Å)	47.661, 76.648, 104.538
α, β, γ (°)	90.000, 90.000, 90.000
Resolution (Å)	1.35 (1.39–1.35)*
R_{merge}	0.052 (1.15)
$I / \sigma I$	16.70 (1.68)
Completeness (%)	98.8 (97.6)
Redundancy	6.5 (6.5)
**Refinement**	
Resolution (Å)	19.77–1.35
No. reflections	83,746
R_{work} / R_{free}	0.184/ 0.200
No. atoms	
Protein	3,031
Water	317
Compound	34
B-factors ($Å^2$)	
protein (chain A)	23.9
compound (chain L)	19.5
Water (chain W)	34.2
R.m.s. deviations	
Bond lengths (Å)/angles (°)	0.010/1.09

*Values in parentheses are for the highest resolution shell.

Appendix Table S2. Binding of CNP520 to and functional affinities for different targets relative to the free plasma concentration at a clinical dose of 85 mg/day

Target<br>Affinity/functional effect	Species	Inhibition at 10 µM (IC_{50})	Ratio of IC_{50} * to free C_{max} in human plasma**
Ghrelin receptor (GHS)	Human	79% (3.2 µM)	83
Lipoxygenase 5-LO	Human	66% (4.81 µM)	122
Imidazoline I2 receptor central	Rat	61% (5.38 µM)	136
Monoamine transporter VMAT2	Rabbit	79% (3.05 µM)	77
Calcium channel L-type, phenylalkylamine site	Rat	78% (1.36 µM)	34
Sodium channel, binding site 2	Rat	104% (0.4 µM)	10
Functional: H Cav 1.2	Human	49.6% (9.1 µM)	231
Functional: H Nav 1.2	Human	23.6% (22.3 µM)	566
Functional: Monoamine transporter VMAT2	Rat	80% (2.74 µM)	69
Dopamine; norepinephrine; serotonin (5HT1A, 5HT2A, 5HT2B); gamma-aminobutyric acid (GABA); acetylcholine; opioid; N methyl-D-aspartic acid (NMDA); cannabinoid (CB1); histamine (H1, H2)		≥30µM	>700

* For the receptor in question

**At a dose of 85 mg (0.048 µM)

Appendix Table S3. Overview of completed clinical studies with CNP520

Study description	Population	No. of Participants exposed to CNP520/ Placebo	Dose/Frequency
**Phase I studies**			
First-in-human, randomized, double-blind, placebo-controlled, single and multiple ascending oral dose study to assess the safety, tolerability, pharmacokinetics and pharmacodynamics of CNP520 in healthy adult and elderly participants	18-55 yrs	38 / 14	Single dose (10, 30, 90, 300, 750, 1125 mg of CNP520 vs placebo)
	≥ 60 yrs	50 / 17	Single dose (10, 90, 300, 750 mg of CNP520 vs placebo)
	≥ 60 yrs	55 / 20	Multiple dose over 14 or 28 days (10, 30, 90, 300 mg q.d.)
	18-55 yrs	55 / 20	Single dose food effect (75 mg)
A randomized, double-blind, placebo-controlled, single ascending and multiple oral dose study, to assess the safety, tolerability, pharmacokinetics and pharmacodynamics of CNP520 in Japanese healthy adult and elderly participants	20-45 yrs	24 / 8	Single-dose (30, 90, 300, 750 mg of CNP520 vs. placebo)
	≥ 60 yrs	8 / 4	Multiple dose (85 mg q.d. vs placebo) over 14 days
An open-label, randomized, single dose cross-over study to assess the relative bioavailability of 3 different CNP520 formulations in healthy adult participants	18-55 yrs	16	Single-dose (10 mg, 50 mg CNP520)
A two part, open-label, two-period, fixed-sequence study in healthy participants to evaluate the pharmaco- kinetics of CNP520 when given alone and in combination with the strong CYP3A4 inhibitor itraconazole or in combination with the strong CYP3A4 inducer rifampicin	18-55 yrs	34	Single-dose (30 mg, 100 mg CNP520)
**Phase IIa study**			

Randomized, double-blind, placebo-controlled, parallel-group study to assess the safety, tolerability, pharmacokinetics and pharmacodynamics of multiple oral doses of CNP520 in healthy elderly participants	≥ 60 yrs	100 / 24	Multiple-dose over 13-weeks (2, 10, 35 or 85 mg q.d. CNP520 vs. placebo)

Appendix Table S4. Study synopsis of completed clinical studies with CNP520

**Title**	A first-in-human, randomized, double-blind, placebo-controlled, single and multiple ascending oral dose study, to assess the safety, tolerability, pharmacokinetics and pharmacodynamics of the BACE inhibitor CNP520 in healthy adults and elderly subjects.
**Brief title**	Study of safety, tolerability, pharmacokinetics and pharmacodynamics of CNP520 in healthy adult and elderly subjects
**Sponsor and Clinical Phase**	Novartis<br>Phase I
**Investigation type**	Drug
**Study type**	Interventional
**Purpose and rationale**	The purpose of this first-in-human study is to assess the safety, tolerability, pharmacokinetics and pharmacodynamics of single and multiple oral ascending doses of CNP520 in Healthy Adults (HA) and Healthy Elderly (HE) subjects and thus to support the future development of CNP520 in Alzheimer's Disease<br>A multi-part study design has been selected since it allows seamless and safe entry into a healthy elderly population which represents the age range of the planned target population (cognitively unimpaired ApoE4 carriers) for development of CNP520. In addition it allows inclusion of study parts dedicated to investigation of pharmacodynamics.
**Primary Objective(s) and Key Secondary Objective**	To determine the safety and tolerability of CNP520 after single oral doses in healthy adult and elderly subjects<br>To determine the safety and tolerability of CNP520 after multiple oral doses in a once daily regimen over 14 days in healthy elderly subjects<br>To assess the effect of single and multiple oral doses of CNP520 on CSF Aβ levels (Aβ1-38; Aβ1-40; Aβ1-42) in healthy elderly subjects
**Secondary Objectives**	To determine the pharmacokinetics of CNP520 after single oral doses in healthy adult and elderly subjects<br>To determine the pharmacokinetics of CNP520 after multiple oral doses (in a once daily regimen over 14 days) in healthy elderly subjects
**Study design**	This is a non-confirmatory FIH, double-blind, randomized, placebo-controlled study of single and multiple ascending oral doses of CNP520 in healthy male and female subjects. The study is planned to be a multicenter study. The study is divided into 2 parts where Part A will have single dose administrations and Part B will have multiple doses administrations.
**Study design (cont.)**	Part A contains 3 distinct parts:<br>• SAD: a single-ascending dose (SAD) study to determine the maximum tolerated dose (MTD), referred to as SAD<br>• SAD PD: a single ascending dose (SAD) study with Pharmacokinetic (PK) and Pharmacodynamic (PD) investigation in CSF and its relation to plasma, referred to as SAD PD

	• SD FE: a single dose (SD) food effect (FE) study to investigate the effect of a high-fat meal on the PK of CNP520 referred to as SD FE.<br><br>SAD and SD FE will be conducted in healthy male adults and SAD PD in healthy elderly male and female of non- child bearing potential.<br><br>Part B is a multiple-ascending dose (MAD) study to determine safety, tolerability, PK and PD in blood and CSF following ascending multiple doses of CNP520 in healthy elderly male and female of non- child bearing potential subjects and referred to as MAD PD.<br><br>Start and execution of SAD, SAD PD and MAD PD will be staggered.<br><br>In SAD, safety and tolerability of the first two dose groups are required to initiate SAD PD in healthy elderly. Once safety and tolerability and PK results from the first dose group of healthy elderly subjects in SAD PD are available, the MAD PD in healthy elderly subjects may start. Knowledge of safety and tolerability and PK data from two dose groups of healthy adults and PK of one dose group of healthy elderly allows comparing the two populations as well as prediction of the steady state PK exposure in Plasma and CSF in healthy elderly subjects. Depending on PK properties of

	CNP520, eg. short t1/2, less PK data may be required before starting the MAD PD part.

Safety, tolerability data from SAD (at least a 3 fold higher dose) as well as pharmacodynamic data from SAD PD are required to choose the appropriate dose for SD FE in healthy adult subjects.

The number of subjects per cohort, number of cohorts, doses ranges and adaptations are described in the table below.

Study part	Number of planned cohorts	Number of subjects per cohort	Minimal envisaged dose	Maximal envisaged dose	Number of additional cohorts	Doses adaptations	Maximal dose
SAD	6	8	10 mg	1125 mg	2	Lower<br>Repeat<br>Intermediate	1125 mg
SAD PD	6<br>Cohort 13 and 14 confirmed	10 (12 in cohort 13 and 14)	10 mg	750 mg	0	Lower<br>Repeat 300 mg in cohort 13 and 750 mg in cohort 14<br>Intermediate	1125 mg
MAD PD	6 (Cohort 20 and 21 condfirmed	12	10 mg/day	300 mg/day	2	Lower<br>Repeat<br>Intermediate<br>Higher	750 mg/day

SD FE is a randomized, single dose, open-label, food effect 2X2 cross-over study in HA. This part will investigate the effect of a high-fat meal on the PK and safety of CNP520. Approximately 10 subjects will be enrolled and will be randomized to receive CNP520 under fed and fasted conditions according to one of the two possible sequences: fed then fasted or fasted then fed.

Population

The study population will be comprised of male healthy adults and male and female (of non-child bearing potential) healthy elderly volunteers. A total of up to approximately 210 subjects may be enrolled to participate in the study, depending on the number of cohorts required and the number of subjects to be replaced. A total of Approximately 138 subjects will be enrolled in Part A: 74 Healthy Adults (64 in SAD, 10 in SD FE) and 64 Healthy Elderly subjects (SAD PD). A total of approximately 72 HE subjects will be enrolled in part B (MAD PD).

SAD and SD FE will be comprised of healthy male adult subjects 18 to 55 years of age and SAD PD and MAD PD will be comprised of healthy elderly male and female subjects of at least 60 years of age.

Inclusion criteria

Inclusion criteria applicable to all subjects

- Written informed consent must be obtained before any assessment is performed.

	**Inclusion criteria applicable to healthy adult subjects**<br>• Healthy male subjects 18 to 55 years of age and in good health<br>• Subjects must weigh at least 50 kg to participate in the study, and must have a body mass index (BMI) within the range of 18 - 30 kg/m^2.<br>**Inclusion criteria applicable to healthy elderly subjects**<br>• Healthy male and postmenopausal female subjects ≥ 60 years of age and in good health<br>• Subjects must weigh at least 50 kg to participate in the study, and must have a body mass index (BMI) within the range of 18 - 32 kg/m^2.<br>• Unless mentioned under exclusion criteria (prohibited medications and foods during the study) regular intake of concomitant medication including but not limited to paracetamol, low dose NSAIDs (except acetylsalicylic acid), lipid lowering drugs, antihypertensive drugs (except for diuretics), vitamins, and hormone replacement therapy is allowed, if on stable treatment for at least 3 months.
**Exclusion criteria**	**Exclusion criteria applicable to all subjects**<br>• Smokers (use of tobacco products in the previous 3 months). Urine cotinine levels will be measured during screening and at each baseline for all subjects. Smokers will be defined as any subject who reports tobacco use and/or who has a urine cotinine ≥ 500 ng/ml<br>• Subjects with presence of diabetes, or other endocrine disorder such as Cushing syndrome.<br>• Subjects with presence and/or a history of thyroid disease or on medications that may alter the thyroid function.<br>• History of neurological disorders including demyelinating diseases.<br>• History of psychiatric disorders.<br>• History/presence of inherited and/or acquired diseases or disorders that may affect the retina<br>• History of disorders related to melanin deficiency<br>• History of head trauma in the past year and/or history of seizures in the past 5 years.<br>• History of drug or alcohol abuse<br><br>**Exclusion criteria applicable to healthy adult subjects**<br>• Sexually active males must use a condom during intercourse while taking drug and for at least 3 months after stopping investigational medication and should not father a child in this period. A condom is required to be used also by vasectomized men in order to prevent delivery of the drug via seminal fluid.<br>• History or presence of impaired renal function as indicated by clinically significantly abnormal creatinine or BUN and/or urea values, or abnormal urinary constituents (e.g., albuminuria).<br>• Use of any prescription drugs, herbal supplements (St. John's Wort included), within four (4) weeks prior to initial dosing, and/or over-the-counter (OTC) medication, dietary supplements (vitamins included) within two (2) weeks prior to initial dosing.<br>**Exclusion criteria applicable to healthy elderly subjects**<br>• Sexually active males must use a condom during intercourse while taking drug and for at least 3 months after stopping investigational medication and should not father a child in this period. A condom is required to be used also by vasectomized men in order to prevent delivery of the drug via seminal fluid.

	• Subjects with back surgery in the past (with the exception of microdiscectomy or laminectomy over 1 level).<br>• History of amnestic cognitive impairment<br>• Subjects with spinal deformities.<br>• Concomitant medications acting on coagulation (particular care to avoid acetylsalicylic acid).<br>• Subjects with elevated Intracranial Pressure detected by Ophthalmoscopy (as part of physical examination done before pre-dose CSF sampling on Day 1).<br>• Clinically significant abnormal PT/aPTT values<br>• History or presence of impaired renal function as indicated by clinically significantly abnormal creatinine or BUN and/or urea values, or abnormal urinary constituents (e.g., albuminuria). Subjects who have an estimated glomerular filtration rate (GFR) <60ml/min(calculated using the Cockcroft-Gault formula) or have 2+ or greater protein on urine dipstick testing at screening and at baseline will be excluded.<br>• Any disability that may prevent the subject from completing all study requirements (e.g., blindness, deafness, communication difficulty).
**Investigational and reference therapy**	Part A<br>Study treatments are defined as following in<br>SAD:<br>Cohort I: single dose of 10 mg CNP520/placebo (1 x 10 mg)<br>Cohort II: single dose of 30 mg CNP520/placebo (3 x 10 mg)<br>Cohort III: single dose of 90 mg CNP520/placebo (5 x 1; 1 x 10; 1 x 75 mg)<br>Cohort IV: single dose of 300 mg CNP520/placebo (4 x 75 mg)<br>Cohort V: single dose of 750 mg CNP520/placebo (10 x 75 mg)<br>Cohort VI: single dose of 1125 mg CNP520/placebo (15 x 75 mg)<br>Cohort VII (as needed) : lower, intermediate, repeat, not higher than 1125 mg CNP520/placebo<br>Cohorts VIII (as needed) : lower, intermediate, repeat, not higher than 1125 mg CNP520/placebo<br>SAD PD<br>Cohort IX: single dose of 10 mg CNP520/placebo (1 x 10 mg)<br>Cohort X: single dose of 90 mg CNP520/placebo (5 x 1; 1 x 10; 1 x 75 mg)<br>Cohort XI: single dose of 300 mg CNP520/placebo (4 x 75 mg)<br>Cohort XII: single dose of 750 mg CNP520/placebo (10 x 75 mg)<br>Cohort XIII: single dose of 300 mg CNP520/placebo (4 x 75 mg)<br>Cohort XIV: single dose of 750 mg CNP520/placebo (10 x 75 mg)<br>SD FE:<br>A: single dose of CNP520 in fasted condition, dose to be determined

	B: single dose of CNP520 in fed condition, dose to be determined<br>All subjects are in Cohort XV randomized to A - B or B - A sequences.<br>Part B Multiple doses, study treatments are defined as the following:<br><br>MAD PD:<br>Cohort XVI: multiple doses of 10 mg CNP520/placebo (1 x 10 mg once daily)<br>Cohort XVII: multiple doses of 30 mg CNP520/placebo (3 x 10 mg once daily)<br>Cohort XVIII: multiple doses of 90 mg CNP520/placebo (5 x 1; 1 x 10; 1 x 75 mg once daily)<br>Cohort XIX: multiple doses of 300 mg CNP520/placebo (4 x 75 mg once daily)<br>Cohort XX: multiple doses of 10 mg CNP520/placebo (1 x 10 mg once daily)<br>Cohort XXI multiple doses of 10 mg CNP520/placebo (1 x 10 mg once daily) for 28 days
**Efficacy assessments**	Aβ in CSF and plasma
**Safety assessments**	• Physical examination, Vital signs, Height and weight, Laboratory evaluation (hematology, clinical chemistry, urinalysis, thyroid function test), ECG, pregnancy, C-SSRS, Neurological examination
**Other assessments**	• PK in CSF, blood and urine<br>• Pharmacogenomics<br>• Optional pharmacogenetics<br>• Other biomarker in CSF, Optional CSF sampling for TREM -2 -/1

**Data analysis**	The primary objective of this study is to evaluate the safety and tolerability of CNP520 after single and/or multiple ascending doses in healthy male and female adult and elderly subjects.<br><br>Summary statistics will be provided for all safety variables as appropriate.<br><br>A secondary objective of this study is to determine the pharmacokinetics of CNP520 after single and multiple oral doses. Descriptive statistics of PK concentrations and parameters in plasma, CSF and urine will be provided by study part and treatment group. In the SAD part, an exploratory assessment of dose proportionality using the power model will be done on Cmax and AUClast and AUcinf in plasma. In the SD FE part, the effect of food on CNP520 on the PK parameters will be assessed by the multiplicative change due to the fed condition. Log transformed PK parameters will be analyzed using a linear fixed effects model. Point estimates and 90%Ci of the ratios of geometric means fed versus fasted will be provided by back transformation on the original scale. No formal inference on PK parameters in CSF or urine is planned.<br><br>Another secondary objective of this study is to assess the effect of single and multiple oral doses of CNP520 on Aβ levels in CSF. They will be analyzed after each cohort of the SDA PD and MAD PD parts. Summary statistics of values, absolute and percent changes from baseline will be provided. The percent change from baseline in Aβ levels will be analyzed using a linear mixed effects model. The model will include treatment (each dose or placebo), time, treatment*time and baseline*time interaction terms, baseline Aβ level as covariate and subject as random effect.
**Key words**	Safety, tolerability, Pharmacokinetics, CSF, healthy adult subjects, healthy elderly subjects, ABeta
**Title**	A randomized, double-blind, placebo-controlled, single ascending and multiple oral dose study, to assess the safety, tolerability, pharmacokinetics and pharmacodynamics of CNP520 in Japanese healthy adult and elderly subjects
**Brief title**	Safety , tolerability and PK/PD assessment after oral doses of CNP520 in Japanese healthy adult and elderly subjects
**Sponsor and Clinical Phase**	Novartis<br><br>Phase I
**Intervention type**	Drug
**Study type**	Interventional
**Purpose and rationale**	This study is designed to evaluate the safety, tolerability, pharmacokinetics and pharmacodynamics of single ascending doses of CNP520 orally administered in Japanese healthy adult and multiple doses in healthy elderly subjects. Comparison with a preceding FIH study should enable the assessment of the effect of Japanese ethnicity on the safety and pharmacokinetic/pharmacodynamic profiles ahead of participation in multinational study to fulfill the local health authority requirement.
**Primary Objective(s)**	To assess the safety and tolerability of CNP520 after single oral doses in Japanese healthy adult subjects<br><br>To assess the safety and tolerability of CNP520 after multiple oral doses in a once daily regimen over 14 days in Japanese healthy elderly subjects

**Secondary Objectives**	To assess pharmacokinetics (PK) of CNP520 after single and multiple oral doses in Japanese healthy adult and elderly subjects<br>To assess the effect of multiple oral doses of CNP520 on CSF Aβ levels (Aβ1-38; Aβ1-40; Aβ1-42) in Japanese healthy elderly subjects
**Study design**	A randomized, double-blind, placebo-controlled, single ascending and multiple oral doses
**Population**	32 of Japanese healthy male subjects aged 20-45 years old inclusive and 12 of Japanese healthy elderly subjects (male and female) aged ≥ 60 years old
**Inclusion criteria**	• Japanese healthy male subjects, aged 20-45 years of age (inclusive) and Japanese healthy elderly subjects ≥60 years old.<br>• Subjects must weigh at least 50 kg to participate in the study, and must have a body mass index (BMI) within the range of 18 to 29 kg/m2 in healthy adults and 18 to 32 kg/m2 in healthy elderly subjects.
**Exclusion criteria**	• Use of other investigational drugs 4 months or 5 half-lives prior to initial dosing, whichever is longer; or longer if required by local regulations.<br>• Score “yes” on item 4 or item 5 of the Suicidal Ideation section of the C-SSRS, if this ideation occurred in the past 6 months, or “yes” on any item of the Suicidal Behavior section, except for the “Non-Suicidal Self-Injurious Behavior” (item also included in the Suicidal Behavior section), if this behavior occurred in the past 2 years<br>• History of psychiatric disorders within 2 years prior to enrollment.<br>• History/presence of inherited and/or acquired diseases or disorders that may affect the retina<br>• History of disorders related to melanin deficiency, e.g. vitiligo.<br>• History of head trauma and/or history of seizures and/or history of severe/significant headache, nausea and vomiting in the last 6 month.
**Investigational and reference therapy**	• CNP520<br>• Placebo
**Efficacy/PD assessments**	• Aβ levels (Aβ1-38; Aβ1-40; Aβ1-42) in CSF and Aβ1-40 in plasma<br>• sAPPα and sAPPβ in CSF
**Safety assessments**	• Physical examination<br>• Neurological examination<br>• C-SSRS<br>• Blood Pressure and Pulse Rate<br>• ECG evaluation<br>• AEs and SAEs
**Other assessments**	• Pharmacokinetics (plasma CNP520, Cmax and AUCs)<br>• Pharmacogenetic assessment
**Data analysis**	No formal statistical hypotheses of the safety or tolerability are to be tested for this study.<br>Summary statistics for safety evaluations such as vital signs, ECG evaluations, standard clinical laboratory evaluations (including immunogenicity) and others will be

	provided by treatment and visit/time. Occurrence of adverse events will be tabulated by preferred term, system organ class and severity for each treatment group and pooled placebo group.
**Key words**	NA

**Title**	An open-label, randomized, single dose cross-over study to assess the relative bioavailability of 3 different CNP520 formulations in healthy adult subjects
**Brief title**	Study of relative bioavailability of 3 different CNP520 formulations in healthy adult subjects
**Sponsor and Clinical Phase**	Novartis<br>Phase I
**Intervention type**	Drug
**Study type**	Interventional
**Purpose and rationale**	The purpose of the study is the following:<br>To assess the relative bioavailability of CNP520 to bridge the clinical service formulation (CSF) used in early Phase I studies and the final market formulation (FMI) to be introduced in Phase II/III studies.<br>Additionally, the bioavailability of FMI and CSF compared to a reference formulation (experimental formulation (EF)) will be evaluated to provide data to establish an in-vitro/in vivo (IVIVC) model.<br>Dose-proportionality for the FMI will also be assessed in order to be as close as possible to and herewith to verify the IVIVC model/correlation for the CSF formulation.
**Primary Objective(s)**	To evaluate the relative bioavailability of a single oral dose of 50 mg CNP520 administered as FMI compared to CSF.
**Secondary Objectives**	To evaluate dose-proportionality of the FMI formulation after single oral dose administration of 10 mg and 50 mg CNP520.<br>To evaluate the relative bioavailability of FMI and CSF compared to EF after single oral dose administration of 50 mg CNP520.<br>To assess the safety and tolerability of single, oral doses of 10 mg and 50 mg CNP520 administered as three different CNP520 formulations (FMI, CSF, and EF) in healthy adult subjects.
**Study design**	This is a non-confirmatory, open-label, randomized, three period, single dose cross-over study in healthy male, adult subjects to assess the relative bioavailability of 3 different CNP520 formulations.<br>A total of 16 subjects will be randomized in a 1:1 ratio into two treatment sequences: Cohort 1 (8 subjects) or Cohort 2 (8 subjects). Screening will occur from Days -28 to Day -2. Baseline 1 will occur on Day -1, Baseline 2 will be on Day 21, and Baseline 3 will be on Day 42.<br>In Treatment Period 1, on Day 1<br>subjects in Cohort 1 will receive CNP520 FMI 50 mg and<br>subjects in Cohort 2 will receive CNP520 CSF 50 mg<br>followed by a 3-week washout period (Days 1 to 21) and Baseline 2 on Day 21.<br>In Treatment Period 2, the order of treatment is reversed, i.e. on Day 22<br>subjects in Cohort 1 will receive CNP520 CSF 50 mg and

	subjects in Cohort 2 will receive CNP520 FMI 50 mg<br>followed by a 3-week washout period (Days 22 to 42), and Baseline 3 on Day 42.<br>At the end of Treatment Period 2, an interim analysis will be performed for data collected in Treatment Periods 1 and 2 while Treatment Period 3 continues.<br>In Treatment Period 3, Cohort 1 and Cohort 2 will be randomized into two parallel subcohorts. On Day 43,<br>subjects in Cohort 1 will be randomized to receive either CNP520 FMI 10 mg (4 subjects) or CNP520 EF 50 mg (4 subjects) and<br>subjects in Cohort 2 will be randomized to receive either CNP520 FMI 10 mg (4 subjects) or CNP520 EF 50 mg (4 subjects),<br>followed by a 3-week washout period (Days 43 to 63).<br>Study completion visit will occur on Day 64 (-1 +2 days).
**Population**	Healthy male subjects aged 18-55. Target enrollment is 16 subjects.
**Inclusion criteria**	Healthy males 18 to 55 years of age in good health as determined by past medical history, physical examination, neurological examination, vital signs, electrocardiogram, and laboratory tests at screening.<br>At screening and Baseline 1 (Day -1), vital signs (systolic and diastolic pressure (BP) and pulse rate) will be assessed in the sitting position after the subject has rested for at least 3 minutes and again after 3 minutes in the standing position. Vital signs should be within the normal range at screening and Baseline 1.<br>Body weight ≥50 kg and a body mass index (BMI) ≥18 to ≤34 kg/m^2. BMI = Body weight (kg) / [Height (m^2)].<br>Ability to communicate well with the investigator, to understand and comply with the requirements of the study.
**Exclusion criteria**	History of hypersensitivity to BACE inhibitors.<br>Clinically significant ECG abnormalities as determined by single 12-lead ECGs and judged by the investigator (if confirmed by single repeat assessment).<br>History of malignancy of any organ system within the past 5 years except for localized tumors not requiring systemic chemo- or radiotherapy such as localized basal cell carcinoma of the skin.<br>History or presence of any clinically significant disease (as judged by the investigator) of any major system organ class including (but not limited to) cardiovascular, pulmonary, metabolic, endocrine, immunological or renal diseases which has not resolved within two weeks prior to initial dosing.<br>Use of any prescription drug, herbal supplements (St.John's Wort included) and/or over the counter (OTC) medication within two (2) weeks prior initial dosing. If needed, (i.e. an incidental and limited need) paracetamol is acceptable, but must be documented in the Concomitant medications/Significant non-drug therapies page of the (e)CRF.<br>History within the last two years of autonomic dysfunction (e.g. recurrent episodes of fainting, palpitations etc.).

	History within the past 2 years or presence of clinically significant neurological or psychiatric disorders including seizures, dementia, head trauma, schizophrenia, major depression, pseudotumor cerebri, bipolar disorder or demyelinating diseases.<br><br>Any medical condition that might lead to or is associated with any cognitive deficit.<br><br>Past or current history of macula degeneration, drusen deposits in retinal pigment epithelium, retina degeneration or mitochondrial diseases/dysfunction as reported by the subject.
**Investigational and reference therapy**	Cohort 1<br>Period 1: FMI 50 mg (n=8)<br>Period 2: CSF 50 mg (n=8)<br>Period 3: FMI 10 mg (n=4); EF 50 mg (n=4)<br>Cohort 2<br>Period 1: CSF 50 mg (n=8)<br>Period 2: FMI 50 mg (n=8)<br>Period 3: FMI 10 mg (n=4); EF 50 mg (n=4)
**Efficacy/PD assessments**	Not applicable.
**Safety assessments**	Physical examination<br>Vital signs<br>Laboratory evaluations (hematology, clinical chemistry, urinalysis<br>Electrocardiogram (ECG)<br>Neurological examination
**Other assessments**	Pharmacokinetic blood collection (CNP520)<br>Exploratory DNA blood collection (optional)
**Data analysis**	Only subjects with evaluable PK parameters data for both test and reference treatment will be included in the primary analysis. Log-transformed PK parameters (Cmax, AUClast, AUCinf) will be analyzed separately using an analysis of variance (ANOVA) model, with fixed effects for sequence, treatment, period, and subject nested within sequence. The point estimate and 90% CI for the difference between the FMI 50 mg (test) and the CSF 50 mg (reference) on the log-scale will be calculated from the model. These values will be back transformed to give a point estimate and 90% CI for the ratio of geometric means.
**Key words**	Relative bioavailability, pharmacokinetics, CNP520, healthy males

**Title**	A two part, open-label, two-period, fixed-sequence study in healthy subjects to evaluate the pharmacokinetics of CNP520 when given alone and in combination with the strong CYP3A4 inhibitor itraconazole or in combination with the strong CYP3A4 inducer rifampin
**Brief title**	Study of pharmacokinetics of CNP520 when given alone and in combination with the strong CYP3A4 inhibitor itraconazole or the strong CYP3A4 inducer rifampin
**Sponsor and Clinical Phase**	Novartis<br>Phase I
**Intervention type**	Drug
**Study type**	Interventional
**Purpose and rationale**	The purpose of conducting a drug-drug interaction (DDI) study with a strong cytochrome P450 3A4 (CYP3A4) inhibitor and a DDI study with a strong CYP3A4 inducer is to provide data relevant for decision-making regarding restrictions of co-medications in future clinical studies and for labeling.
**Primary Objective(s)**	• To evaluate the effect of the strong CYP3A4 inhibitor Itraconazole on the pharmacokinetics (PK) of CNP520 following administration of single oral doses of CNP520.<br>• To evaluate the effect of the strong CYP3A4 inducer Rifampin on the PK of CNP520 following administration of single oral doses of CNP520.
**Secondary Objectives**	• To assess the safety and tolerability of single oral doses of CNP520 when given alone or in combination with multiple, oral doses of Itraconazole or Rifampin in healthy subjects.<br>• To assess the plasma exposure of Itraconazole, OH-itraconazole and Rifampin when given in combination with CNP520.
**Study design**	This is a confirmatory, two part, open-label, two-period, fixed-sequence study in healthy subjects to evaluate the PK of CNP520 when given alone and in combination with the strong CYP3A4 inhibitor Itraconazole or in combination with the strong CYP3A4 inducer Rifampin. Different subjects will be enrolled in each part.<br>**Part 1** will evaluate the effect of the strong CYP3A4 inhibitor Itraconazole on the PK and safety of a single oral dose of CNP520 in healthy volunteers (n = 18) in an open-label, two-period, fixed-sequence study. Screening will occur from Day -28 to Day -2. Baseline 1 will occur on Day -1 and Baseline 2 will occur on Day 14. Subjects will receive a single dose of CNP520 30 mg (three 10 mg hard gelatin capsules) on Day 1 and Day 19 0.5 h after completion of a standardized breakfast. A minimum wash out period of 18 days between the first and second dose of CNP520 is required. Itraconazole 200 mg (two 100 mg capsules) will be given once daily in a fed state (given immediately after completion of a standardized breakfast) starting on Day 15 (4 days before the CNP520 dose on Day 19) until Day 30 (the day before the last PK sample collection). On Day 19, Itraconazole will be given approximately 0.5 h before administration of the CNP520 dose. Subjects will remain in clinic for 48 hours post-dose of CNP520 (Period 1: Days 1-3; Period 2: Days 19-21). Study completion visit is Day 37 (+ 1 Day). PK samples will be collected for determination of CNP520 (Period 1:

	Days 1-13; Period 2: Days 19-31), Itraconazole and its metabolite hydroxy-itraconazole (Period 2: Days 17, 19, 20, 21, 22, 25, 28, and 31).<br><br>**Part 2** will evaluate the effect of the strong CYP3A4 inducer Rifampin on the PK and safety of a single oral dose of CNP520 in healthy volunteers (n=16) in an open-label, two-period, fixed-sequence study. Screening will occur from Day -28 to Day -2. Baseline 1 will occur on Day -1 and Baseline 2 will occur on Day 10. Subjects will receive a single dose of CNP520 100 mg (75 mg and 25 mg hard gelatin capsules) on Day 1 and Day 18 in a fasted state. A standardized breakfast will be served 2 h after intake of CNP520. A minimum wash out period of 17 days between the first and second dose of CNP520 is required. Rifampin 600 mg (two 300 mg capsules) will be given once daily in the fasted state starting on Day 11 (7 days before the CNP520 dose on Day 18) until Day 25 (the day before the last PK sample collection). In Period 2, Day 18, CNP520 and Rifampin will be co-administered. Subjects will remain in clinic for 48 hours post-dose of CNP520 (Period 1: Days 1-3; Period 2: Days 18-20). Study completion visit is Day 33 (+ 1 Day). PK samples will be collected for determination of CNP520 (Period 1: Days 1-9; Period 2: Days 18-26) and Rifampin (Period 2: Days 17, 18, 19, and 26).
**Population**	Healthy male subjects aged 18-55. Target enrollment for Part 1 is 18 subjects and for Part 2 is 16 subjects.
**Inclusion criteria**	• Written informed consent.<br>• Healthy males 18 to 55 years of age in good health as determined by past medical history, physical examination, neurological examination, vital signs, electrocardiogram, and laboratory tests at screening. Each subject is only eligible for partcipation in one part of the study.<br>• At screening and first baseline (Day -1), vital signs (systolic and diastolic pressure (BP) and pulse rate) will be assessed in the sitting position after the subject has rested for at least 3 minutes and again after 3 minutes in the standing position. Vital signs should be within the normal range at screening and first baseline.<br>• Body weight ≥50 kg and a body mass index (BMI) ≥18 to ≤34 kg/m2. BMI = Body weight (kg) / [Height (m2)].<br>• Ability to communicate well with the investigator, to understand and comply with the requirements of the study.
**Exclusion criteria**	• History of hypersensitivity to BACE inhibitors.<br>• Clinically significant ECG abnormalities as determined by single 12-lead ECGs and judged by the investigator (if confirmed by single repeat assessment).<br>• History of malignancy of any organ system within the past 5 years except for localized tumors not requiring systemic chemo- or radiotherapy such as localized basal cell carcinoma of the skin.<br>• History or presence of any clinically significant disease (as judged by the investigator) of any major system organ class including (but not limited to) cardiovascular, pulmonary, metabolic, endocrine, immunological or renal diseases which has not resolved within two weeks prior to initial dosing.<br>• Use of any prescription drugs, herbal supplements (St. John’s Wort included), within four (4) weeks prior to initial dosing, and/or over-the-

	counter (OTC) medication, dietary supplements (vitamins included) within two (2) weeks prior to initial dosing. If needed, (i.e. an incidental and limited need) paracetamol, is acceptable, but must be documented in the Concomitant medications / Significant non-drug therapies page of the (e)CRF.<br>• History within the last two years of autonomic dysfunction (e.g. recurrent episodes of fainting, palpitations etc.).<br>• History within the past 2 years or presence of clinically significant neurological or psychiatric disorders including seizures, dementia, head trauma, schizophrenia, major depression, pseudotumor cerebri, bipolar disorder or demyelinating diseases.<br>• Any medical condition that might lead to or is associated with any cognitive deficit.<br>• Past or current history of macula degeneration, drusen deposits in retinal pigment epithelium, retina degeneration or mitochondrial diseases/dysfunction as reported by the subject.
**Investigational and reference therapy**	Part 1<br>• CNP520 30 mg<br>• Itraconazole 200 mg<br>Part 2<br>• CNP520 100 mg<br>• Rifampin 600 mg
**Efficacy/PD assessments**	Not applicable.
**Safety assessments**	• Physical examination<br>• Vital signs<br>• Laboratory evaluations (hematology, clinical chemistry, urinalysis)<br>• Electrocardiogram (ECG)<br>• Neurological examination<br>• C-SSRS
**Other assessments**	• Pharmacokinetic blood collection: CNP520<br>• Pharmacokinetic blood collection: Itraconazole<br>• Pharmacokinetic blood collection: Rifampin<br>• Exploratory DNA blood collection (optional)
**Data analysis**	In Part 1, log transformed CNP520 primary PK parameters (Cmax, AUClast and AUCinf) will be analyzed by an ANOVA model with a fixed effect for treatment and subject. Point estimates and 90% confidence intervals for the ratios of the treatment means CNP520 plus Itraconazole (test) vs. CNP520 alone (reference) will be provided for all PK parameters by back transformation to the original scale.<br>The same method of analysis will be used for Part 2 (CYP3A4 inducer Rifampin).
**Key words**	Drug-drug interaction, pharmacokinetics, CNP520, CYP3A4, Itraconazole, Rifampin, healthy males

**Title**	A randomized, double-blind, placebo-controlled, parallel-group study to assess the safety, tolerability, pharmacokinetics and pharmacodynamics of multiple oral doses of CNP520 in healthy elderly subjects
**Brief title**	Dose-ranging safety and tolerability study in subjects ≥60 years of age
**Sponsor and Clinical Phase**	Novartis<br>Phase II
**Intervention type**	Drug
**Study type**	Interventional
**Purpose and rationale**	The primary purpose of this study is to determine the safety of CNP520 over 3 months to allow initiation future long-term efficacy trials in patients with Alzheimer's disease (AD) or in subjects at risk of AD. In addition, data relevant for Pharmacokinetic/Pharmacodynamic modeling will be obtained in order to define the target dose in subsequent efficacy studies.
**Primary Objective(s)**	To determine the safety and tolerability of multiple, once-daily oral doses of CNP520 over 13 weeks in healthy elderly subjects
**Secondary Objectives**	• To assess the change from baseline of CSF Aβ concentrations (Aβ1-38; Aβ1-40; Aβ1-42) in healthy elderly subjects over 13 weeks treatment with CNP520 as compared to placebo<br>• To determine the CNP520 plasma PK and CSF concentrations of once daily oral doses of CNP520 over 13 weeks in healthy elderly subjects

**Study design**	This randomized, double-blind, placebo-controlled, study, has a parallel-group design. Once-daily oral doses of CNP520 will be given over 13 weeks duration to healthy elderly subjects aged 60 to 80 years. This is a non-confirmatory study that is planned to be conducted at multiple sites worldwide.<br><br>The study will consist of an up to 28-day screening period, a 2 day baseline period including a cerebrospinal fluid sample collection, and 13 week treatment period followed by a Study Completion evaluation approximately 4 weeks after the last drug administration. Approximately 125 subjects will be randomized to five treatment groups, four active groups and one placebo. The randomization ratio will be 1:1:1:1:1. Approximately 25 subjects are planned to be enrolled per treatment group.<br><br>Subjects will come for site visits every two weeks during the treatment period and will be given study drug to take at home for the interval up to the next scheduled visit (2 weeks).<br><br>There will be 3 lumbar punctures per subject:<br>• A baseline lumbar puncture<br>• A second lumbar puncture at one of the following weeks (2 weeks (V6) or 4 weeks (V7) or 6 weeks (V8) or 8 weeks (V9) or 10 weeks (V10), this will be assigned per the Sponsor<br>• A third lumbar puncture is scheduled 24 hours after the last drug administration.<br><br>Safety, pharmacokinetic and pharmacodynamic assessments are performed during the course of the study. Safety assessments will include physical examinations, ECGs, vital signs, standard clinical laboratory evaluations (hematology, blood chemistry, urinalysis), Neurological examination, Cognitive assessments, C-SSRS, ophthalmological assessments (Visual Field Test and Best corrected Visual acuity), dermatological assessment, adverse event and serious adverse event monitoring.
**Population**	The study population will be comprised of healthy male and female subjects aged 60 to 80 years. A total of approximately 125 subjects will be enrolled in the study and randomized and approximately 100 subjects are expected to complete the study.
**Main inclusion criteria**	• Male and postmenopausal female subjects aged 60 to 80 years, and in good health as determined by past medical history, physical examination, vital signs, electrocardiogram, and laboratory tests<br>• Subjects must weigh at least 45 kg; (BMI) within the range of ≥18 to ≤34 kg/m2.<br>• Total MMSE score ≥25 for subjects with low educational attainment or ≥27 for subjects with high educational attainment<br>• Ability to perform cognitive assessments

**Main exclusion criteria**	• History or presence of any clinically significant disease (as judged by the investigator) of any major system organ class incl. (but not limited to) cardiovascular, pulmonary, metabolic, endocrine, immunological or renal diseases which has not resolved within two weeks prior to initial dosing.<br>• Heavy smokers<br>• Use of drugs known to be strong inhibitors or inducers of CYP3A4 or narrow-therapeutic index drugs known to be primarily metabolized by CYP2C or CYP3A isoenzymes<br>• History /presence of clinically significant neurological or psychiatric disorders<br>• History or presence of severely impaired renal function as indicated by an estimated glomerular filtration rate (GFR) <30ml/min<br>• Subjects with known genetic deficiency of melanin pigment production (i.e. albinism)
**Investigational and reference therapy**	Part I<br>• Placebo o.d.<br>• CNP520 2 mg o.d.<br>• CNP520 10 mg o.d.<br>• CNP520 35 mg o.d.<br>• CNP520 85 mg o.d.
**Main Safety assessments**	• Adverse event data<br>• ECG, vital signs and laboratory data<br>• Assessment of cognitive function (Part I only)<br>• Dermatological and ophthalmological assessments (visual field/acuity)<br>• C-SSRS
**Main Efficacy/PD assessments**	No efficacy assessments<br>Pharmacodynamic assessments include quantification of amyloid Beta in cerebrospinal fluid (CSF)
**Main PK assessments**	Quantification of CNP520 in plasma and CSF

**Data analysis**	Safety and tolerability: Data will be listed and summarized using descriptive statistics.<br><br>Pharmacodynamics: Summary statistics of absolute CSF Aβ concentrations will be provided by treatment and visit. Percent changes from baseline at week 13 will be analyzed using an ANCOVA model including the baseline value as covariate and treatment as factor. The estimated differences between each CNP520 dose and placebo and their 90% CI will be provided.<br><br>Pharmacokinetics: Descriptive statistics of CNP520 concentrations and parameters in plasma and of concentration in CSF will be provided.<br><br>PK/PD modeling will be also performed in order to define the dose to be used in further studies.
**Key words**	Amyloid-β; cerebrospinal fluid; Alzheimer's Disease; PK/PD modeling

Appendix Table S5. Incidence of AEs by primary system organ class (SOC) after single dose in healthy participants ≥ 60 years, arranged in descending order of total frequency

	**Placebo n (%)**	**CNP520 10mg n (%)**	**CNP520 90mg n (%)**	**CNP520 300mg n (%)**	**CNP520 750mg n (%)**	**Total n (%)**
Participants exposed	N = 13	N = 6	N = 6	N = 8	N = 9	N = 42
Participants with at least one AE	9 (69.2)	5 (83.3)	3 (50.0)	6 (75.0)	8 (88.9)	31 (73.8)
Musculoskeletal and connective tissue disorders	3 (23.1)	3 (50.0)	3 (50.0)	6 (75.0)	6 (66.7)	21 (50.0)
Nervous system disorders	4 (30.8)	2 (33.3)	1 (16.7)	2 (25.0)	3 (33.3)	12 (28.6)
General disorders and administration site conditions	2 (15.4)	1 (16.7)	0 (0.0)	0 (0.0)	1 (11.1)	4 (9.5)
Injury, poisoning and procedural complications	1 (7.7)	0 (0.0)	0 (0.0)	0 (0.0)	3 (33.3)	4 (9.5)
Gastrointestinal disorders	1 (7.7)	1 (16.7)	0 (0.0)	0 (0.0)	2 (22.2)	4 (9.5)
Respiratory, thoracic and mediastinal disorders	2 (15.4)	0 (0.0)	0 (0.0)	0 (0.0)	0 (0.0)	2 (4.8)
Infections and infestations	1 (7.7)	0 (0.0)	0 (0.0)	0 (0.0)	0 (0.0)	1 (2.4)

Metabolism and nutrition disorders	0 (0.0)	1 (16.7)	0 (0.0)	0 (0.0)	0 (0.0)	1 (2.4)
Ear and labyrinth disorders	0 (0.0)	0 (0.0)	0 (0.0)	1 (12.5)	0 (0.0)	1 (2.4)
Eye disorders	0 (0.0)	0 (0.0)	1 (16.7)	0 (0.0)	0 (0.0)	1 (2.4)
Psychiatric disorders	0 (0.0)	0 (0.0)	1 (16.7)	0 (0.0)	0 (0.0)	1 (2.4)
Vascular disorders	0 (0.0)	0 (0.0)	1 (16.7)	0 (0.0)	0 (0.0)	1 (2.4)

Appendix Table S6. Incidence of AEs by primary system organ class (SOC) after single dose in healthy participants ≥ 60 years, arranged in descending order of total frequency

	**Placebo n (%)**	**CNP520 300mg n (%)**	**CNP520 750mg n (%)**	**Total n (%)**
Participants exposed	N = 4	N = 10	N = 11	N = 25
Participants with at least one AE	4 (100.0)	10 (100.0)	9 (81.8)	23 (92.0)
Nervous system disorders	3 (75.0)	4 (40.0)	7 (63.6)	14 (56.0)
Musculoskeletal and connective tissue disorders	3 (75.0)	2 (20.0)	5 (45.5)	10 (40.0)
Gastrointestinal disorders	0 (0.0)	4 (40.0)	3 (27.3)	7 (28.0)
General disorders and administration site conditions	0 (0.0)	3 (30.0)	3 (27.3)	6 (24.0)
Skin and subcutaneous tissue disorders	1 (25.0)	3 (30.0)	1 (9.1)	5 (20.0)
Respiratory, thoracic and mediastinal disorders	0 (0.0)	2 (20.0)	0 (0.0)	2 (8.0)
Blood and lymphatic system disorders	0 (0.0)	1 (10.0)	0 (0.0)	1 (4.0)
Cardiac disorders	0 (0.0)	1 (10.0)	0 (0.0)	1 (4.0)
Infections and infestations	0 (0.0)	0 (0.0)	1 (9.1)	1 (4.0)
Renal and urinary disorders	0 (0.0)	0 (0.0)	1 (9.1)	1 (4.0)

Appendix Table S7. Incidence of AEs by primary system organ class (SOC) after multiple, once-daily dosing over two weeks in healthy participants ≥ 60 years, arranged in descending order of total frequency

	Placebo n (%)	CNP520 10 mg n (%)	CNP520 30 mg n (%)	CNP520 90 mg n (%)	CNP520 300 mg n (%)	Total n (%)
Participants exposed	N = 16	N = 9	N = 9	N = 9	N = 8	N = 51
Participants with any AE(s)	9 (56.3)	6 (66.7)	5 (55.6)	6 (66.7)	6 (75.0)	32 (62.7)
Gastrointestinal disorders	5 (31.3)	3 (33.3)	2 (22.2)	3 (33.3)	2 (25.0)	15 (29.4)
Nervous system disorders	6 (37.5)	4 (44.4)	0 (0.0)	1 (11.1)	3 (37.5)	14 (27.5)
Infections and infestations	0 (0.0)	2 (22.2)	2 (22.2)	2 (22.2)	0 (0.0)	6 (11.8)
Musculoskeletal and connective tissue disorders	2 (12.5)	1 (11.1)	2 (22.2)	0 (0.0)	0 (0.0)	5 (9.8)
Renal and urinary disorders	1 (6.3)	0 (0.0)	0 (0.0)	1 (11.1)	1 (12.5)	3 (5.9)
Respiratory, thoracic and mediastinal disorders	0 (0.0)	1 (11.1)	0 (0.0)	1 (11.1)	0 (0.0)	2 (3.9)
Skin and subcutaneous tissue disorders	0 (0.0)	0 (0.0)	0 (0.0)	0 (0.0)	2 (25.0)	2 (3.9)
Eye disorders	0 (0.0)	1 (11.1)	0 (0.0)	1 (11.1)	0 (0.0)	2 (3.9)
General disorders and administration site conditions	0 (0.0)	0 (0.0)	1 (11.1)	0 (0.0)	1 (12.5)	2 (3.9)
Metabolism and nutrition disorders	0 (0.0)	0 (0.0)	0 (0.0)	0 (0.0)	2 (25.0)	2 (3.9)
Ear and labyrinth disorders	0 (0.0)	0 (0.0)	1 (11.1)	0 (0.0)	0 (0.0)	1 (2.0)
Psychiatric disorders	0 (0.0)	1 (11.1)	0 (0.0)	0 (0.0)	0 (0.0)	1 (2.0)

Appendix Table S8. Incidence of adverse events after 3 months daily dosing in healthy participants ≥ 60 years, by primary system organ class, arranged in descending order of frequency (in total group)

System organ class	Placebo<br>n (%)	CNP520<br>2 mg<br>n (%)	CNP520<br>10 mg<br>n (%)	CNP520<br>35 mg<br>n (%)	CNP520<br>85 mg<br>n (%)	CNP520<br>all doses<br>n (%)
Participants exposed	N = 24	N = 25	N = 25	N = 26	N = 24	N = 100
Participants with at least one AE	18 (75.0)	19 (76.0)	22 (88.0)	21 (80.8)	18 (75.0)	80 (80.0)
Nervous system disorders	10 (41.7)	13 (52.0)	10 (40.0)	9 (34.6)	8 (33.3)	40 (40.0)
Injury, poisoning and procedural complications	10 (41.7)	7 (28.0)	11 (44.0)	10 (38.5)	8 (33.3)	36 (36.0)
Gastrointestinal disorders	8 (33.3)	7 (28.0)	9 (36.0)	9 (34.6)	4 (16.7)	29 (29.0)
Musculoskeletal and connective tissue disorders	10 (41.7)	7 (28.0)	7 (28.0)	8 (30.8)	5 (20.8)	27 (27.0)
Infections and infestations	7 (29.2)	3 (12.0)	10 (40.0)	6 (23.1)	4 (16.7)	23 (23.0)
Respiratory, thoracic and mediastinal disorders	7 (29.2)	6 (24.0)	6 (24.0)	5 (19.2)	5 (20.8)	22 (22.0)
Skin and subcutaneous tissue disorders	1 (4.2)	3 (12.0)	7 (28.0)	2 (7.7)	6 (25.0)	18 (18.0)
General disorders and administration site conditions	5 (20.8)	1 (4.0)	2 (8.0)	4 (15.4)	5 (20.8)	12 (12.0)
Eye disorders	2 (8.3)	2 (8.0)	5 (20.0)	0 (0.0)	4 (16.7)	11 (11.0)
Psychiatric disorders	3 (12.5)	1 (4.0)	2 (8.0)	1 (3.8)	2 (8.3)	6 (6.0)
Investigations	1 (4.2)	2 (8.0)	0 (0.0)	1 (3.8)	1 (4.2)	4 (4.0)
Renal and urinary disorders	0 (0.0)	0 (0.0)	2 (8.0)	2 (7.7)	1 (4.2)	5 (5.0)
Ear and labyrinth disorders	0 (0.0)	0 (0.0)	1 (4.0)	1 (3.8)	1 (4.2)	3 (3.0)
Reproductive system and breast disorders	1 (4.2)	0 (0.0)	2 (8.0)	0 (0.0)	0 (0.0)	2 (2.0)
Vascular disorders	0 (0.0)	0 (0.0)	2 (8.0)	0 (0.0)	1 (4.2)	3 (3.0)
Metabolism and nutrition disorders	0 (0.0)	1 (4.0)	1 (4.0)	0 (0.0)	0 (0.0)	2 (2.0)
Neoplasms benign, malignant & unspecified (incl. cysts and polyps)	0 (0.0)	1 (4.0)	1 (4.0)	0 (0.0)	0 (0.0)	2 (2.0)
Blood and lymphatic system disorders	0 (0.0)	0 (0.0)	0 (0.0)	1 (3.8)	0 (0.0)	1 (1.0)
Immune system disorders	0 (0.0)	0 (0.0)	0 (0.0)	0 (0.0)	1 (4.2)	1 (1.0)

Appendix Table S9. Plasma PK parameters following single dose administration of CNP520 to healthy adult participants, to healthy participants ≥ 60 years and to healthy adult participants following intake in fed and fasted conditions*

**Study (n)**	**C_{max} (ng/ml)**	**T_{max} (h)**	**AUC_{inf} (h*ng/ml)**	**$T_{1/2}$ (h)**
**Single dose adults**				
10 mg (6)	20.9 (18.9)	4.00 (2.00–6.00)	948 (23.9)[1)]	61.3(19.7)[1)]
30 mg (12)	65.7 (25.2)	3.50 (2.00–6.00)	3070 (21.9)	64.3 (18.4)
90 mg (6)	138 (17.8)	3.50 (3.00–4.00)	7190 (14.4)	66.2 (10.5)
300 mg (6)	498 (46.1)	6.00 (2.00–8.00)	23500 (40.9)	57.9 (21.8)
750 mg (6)	1150 (45.7)	2.50 (1.00–6.00)	90100 (30.6)	83.8 (21.5)
1250 mg (2)[2)]	(2850;2890)	(4.00;8.00)	(168000;184000)	(76.4;86.1)
**Single dose adults ≥ 60 years**	**C_{max} (ng/ml)**	**T_{max} (h)**		
10 mg (6)	16.0 (20.4)	4.02 (2.00–6.03)		
90 mg (6)	172 (43.2)	2.02 (2.02–6.02)		
300 mg (18)	440 (41.9)	5.04 (2.00–24.0)		
750 mg (20)	1120 (57.8)	3.00 (2.00–34.0)		
**Food effect adults**	**C_{max} [3)] (ng/ml)**	**Tmax (h)**	**$AUC_{0–72h}$ [4)] (h*ng/ml)**	
75 mg Fed (10)	179 (23.8)	4.04 (3.00–8.00)	4870 (23.7)	not determined
75 mg Fasted (10)	164 (31.5)	3.50 (1.50–8.00)	4450 (27.5)	not determined

*Results are presented as mean (%CV), except for T_{max} presented as median (min–max).

1) (n = 5)
2) Individual data points presented
3) Ratio (90% Confidence intervaI) for geometric LS mean C_{max} Fed *vs* Fasted: 1.11 (0.89–1.38)
4) Ratio (90% Confidence intervaI) for geometric LS mean $AUC_{0–72h}$ Fed *vs* Fasted 1.10 (0.99–1.23)

Appendix Table S10. CNP520 Plasma PK parameters following multiple dose administration to healthy participants ≥ 60 years for 14 days (n = 6–8) and 3 months (n = 22–24)*

Administration period, dose	C_{max} (ng/ml)	T_{max} (h)	$AUC_{0–24h}$ (h*ng/ml)	Mean concentration[1)] (nM)	Accumulation ratio[2)]
**14 days Day 14**					
10 mg	84.3 (29.6)	2.53 (2.00 - 4.02)	1400 (30.0)	114	4.44 (19.0)
30 mg	211 (35.8)	3.00 (2.02 - 4.00)	3580 (32.0)	292	4.10 (13.8)
90 mg	581 (22.2)	3.00 (2.00 - 3.02)	9380 (20.0)	762	3.72 (19.3)
300 mg	1710 (25.1)	2.51 (2.00 - 4.00)	28300 (22.9)	2312	4.34 (29.6)
**3 months Day 91**					
2 mg	16.6 (33.1)	2.50 (0 - 12.1)	313 (37.5)	25.6	5.86 (38.4)
10 mg	81.0 (36.1)	2.50 (0- 12.5)	1500 (31.7)	123	5.33 (19.7)
35 mg	237 (27.7)	2.50 (0 - 12.0)	4450 (24.5)	371	4.75 (24.5)
85 mg	602 (25.0)	2.50 (0 - 12.0)	11200 (29.7)	910	5.02 (29.3)

*Results are presented as mean (CV%), except for T_{max} presented as median (min–max).

1) Mean concentration was obtained by dividing the AUC by 24 hours, and converting to nM using the molecular weight of 513.8.
2) Accumulation ratio is calculated as multiple dose AUC 0–24h/single dose $AUC_{0–24h}$

Appendix Table S11. CSF PK parameters in healthy participants ≥ 60 years of age following single dose administration of CNP520 (n = 6–8), following 14 days multiple dose administration of CNP520 (n = 6–9) and following 3 months multiple dose administration of CNP520 (n = 22–24)*

**Single dose adults ≥ 60 years**	**C_{max} (ng/ml)**	**Ratio AUC CSF/AUC plasma**
10 mg	< LLOQ	NC**
90 mg	3.33 (65.2)	0.02 (56.7)
300 mg	10.5 (49.1)	0.02 (14.9)
750 mg	20.4 (44.8)	0.02 (31.2)
**2 weeks multiple dose adults ≥ 60 years**	**CSF conc (ng/ml) 24h after dose Day 14**	**CSF/plasma conc (ng/ml) 24h after dose Day 14**
10 mg q.d.	< LLOQ	NC
30 mg q.d.	3.33 (41.0)	0.02 (32)
90 mg q.d.	9.48 (26.7)	0.03 (19)
300 mg q.d.	29.8 (14.4)	0.03(16)
**3 month study adults ≥ 60 years**	**CSF conc (ng/ml) 24h after dose Day 91**	**CSF/plasma conc (ng/ml) 24h after dose Day 91**
2 mg q.d.	0.305 (32.5)	0.03 (21.5)
10 mg q.d.	1.44 (29.8)	0.03 (20.0)
35 mg q.d.	4.52 (20.9)	0.03 (15.1)
85 mg q.d.	10.4 (31.4)	0.03 (18.4)

*Results are presented as mean (coefficient of variation %)

**NC, Not calculated

Babin AL, Cannet C, Gerard C, Saint-Mezard P, Page CP, Sparrer H, Matsuguchi T, Beckmann N (2012) Bleomycin-induced lung injury in mice investigated by MRI: model assessment for target analysis. Magnetic resonance in medicine 67**:** 499-509

Egger C, Cannet C, Gerard C, Jarman E, Jarai G, Feige A, Suply T, Micard A, Dunbar A, Tigani B et al (2013) Administration of bleomycin via the oropharyngeal aspiration route leads to sustained lung fibrosis in mice and rats as quantified by UTE-MRI and histology. PloS one 8**:** e63432

Emsley P, Lohkamp B, Scott WG, Cowtan K (2010) Features and development of Coot. Acta Crystallogr D Biol Crystallogr 66**:** 486-501

Hanessian S, Yun H, Hou Y, Yang G, Bayrakdarian M, Therrien E, Moitessier N, Roggo S, Veenstra S, Tintelnot-Blomley M et al (2005) Structure-based design, synthesis, and memapsin 2 (BACE) inhibitory activity of carbocyclic and heterocyclic peptidomimetics. Journal of medicinal chemistry 48**:** 5175-5190

Neumann U, Rueeger H, Machauer R, Veenstra SJ, Lueoend RM, Tintelnot-Blomley M, Laue G, Beltz K, Vogg B, Schmid P et al (2015) A novel BACE inhibitor NB-360 shows a superior pharmacological profile and robust reduction of amyloid-beta and neuroinflammation in APP transgenic mice. Molecular neurodegeneration 10**:** 44

Rahier J, Stevens M, de Menten Y, Henquin JC (1989) Determination of antigen concentration in tissue sections by immunodensitometry. Laboratory investigation; a journal of technical methods and pathology 61**:** 357-363

Ruifrok AC, Johnston DA (2001) Quantification of histochemical staining by color deconvolution. Analytical and quantitative cytology and histology 23**:** 291-299
