# Supplementary material for: The BACE‐1 inhibitor CNP520 for prevention trials in Alzheimer's disease
Source: EMBO Mol Med. 2018 Sep 17;10(11):e9316. doi: 10.15252/emmm.201809316 (PMC6220303; doi:10.15252/emmm.201809316)
Supplement: Supplementary file 2 — Expanded View Figures PDF [file EMMM-10-e9316-s002.pdf]

Expanded View Figures

A

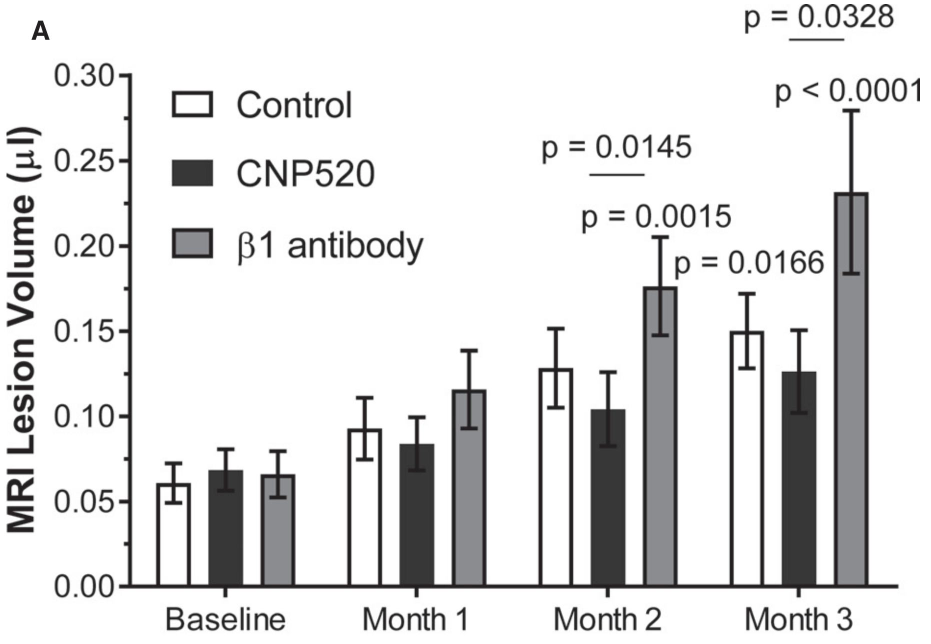

B

	control	CNP520	β1 antibody
animals / group	19	15	18
Hemosiderin (Perl's staining)			
Grade 1	9	7	18
Grade 2	1	0	0
% affected	53	47	100
Hemorrhage (H & E staining)			
Grade 1	6	1	5
Grade 2	0	0	1
% affected	32	7	33

Figure EV1. Assessment of intracerebral micro-hemorrhages in APP23-transgenic mice, dosed orally with CNP520 at 30 mg/kg for 3 months.

A Assessment of brain lesion volume by longitudinal magnetic resonance imaging. For description of the method, see Beckmann *et al* (2016). White bars show the APP23-typical age-dependent increase in lesion volume, and black bars show the effect of CNP520. The antibody β1 serves as positive control and shows enhancement of lesion volume over time. ANOVA test with random effects was applied. Shown is average ± SEM; for *n* numbers, see table in panel (B). Non-underlined *P*-values: comparison to baseline, only significant differences are shown. Underlined *P*-values: significant differences between treatment groups at the given time point.

B Results of histopathological staining using Perl's staining and H&E staining. APP23 (21 months of age) was sacrificed; four brain sections were processed and stained with hematoxylin and eosin (H&E) and Prussian blue for hemosiderin deposits. Levels 2, 3, and 4, and one additional slide at hypothalamus level (level 2'). Brain sections stained with hematoxylin and eosin and with Perl's Prussian blue were examined under a light microscope. The distribution, size, and staining intensity of hemosiderin deposits in Perl's Prussian blue-stained sections were assessed and recorded. Table lists the number of animals affected, and the percentage.

© 2018 Novartis AG

Figure EV2. Effect of CNP520 on microglia and astrocytes in APP23 mice.
Mice were dosed with CNP520 in food pellets. Low dose: 0.03 g CNP520/kg food, exposure corresponds to a daily oral dose of 4 mg/kg. High dose: 0.3 g/kg food, exposure corresponds to a daily oral dose of 40 mg/kg. Half brains were embedded in paraffin, cut, and stained with Iba1 antibody for activated microglia and with GFAP antibody for astrocytes.

A Plaque-associated area of Iba1-positive cells.
B Non-plaque-associated Iba1-positive cells.
C Correlation of normalized plaque-associated Iba1 area with normalized plaque area. Shown is average ± SEM; dashed lines show the 95% confidence interval.
D Correlation of normalized non-plaque-associated area with normalized plaque area. Shown is average ± SEM; dashed lines show the 95% confidence interval.
E Plaque-associated area of GFAP-positive cells.
F Non-plaque-associated GFAP-positive cells.
G Correlation of normalized plaque-associated GFAP-positive area with normalized plaque area. Shown is average ± SEM; dashed lines show the 95% confidence interval.
H Correlation of normalized non-plaque-associated area with normalized plaque area. Shown is average ± SEM; dashed lines show the 95% confidence interval.

Data information: (A and B) $n = 7$/group. (E and F) Baseline, $n = 10$, vehicle: $n = 17$, CNP520 low dose: $n = 14$, CNP520 high dose: $n = 13$. One way ANOVA with Dunnett's multiple comparison test was used in panels (A, B, E, and F).

 © 2018 Novartis AG

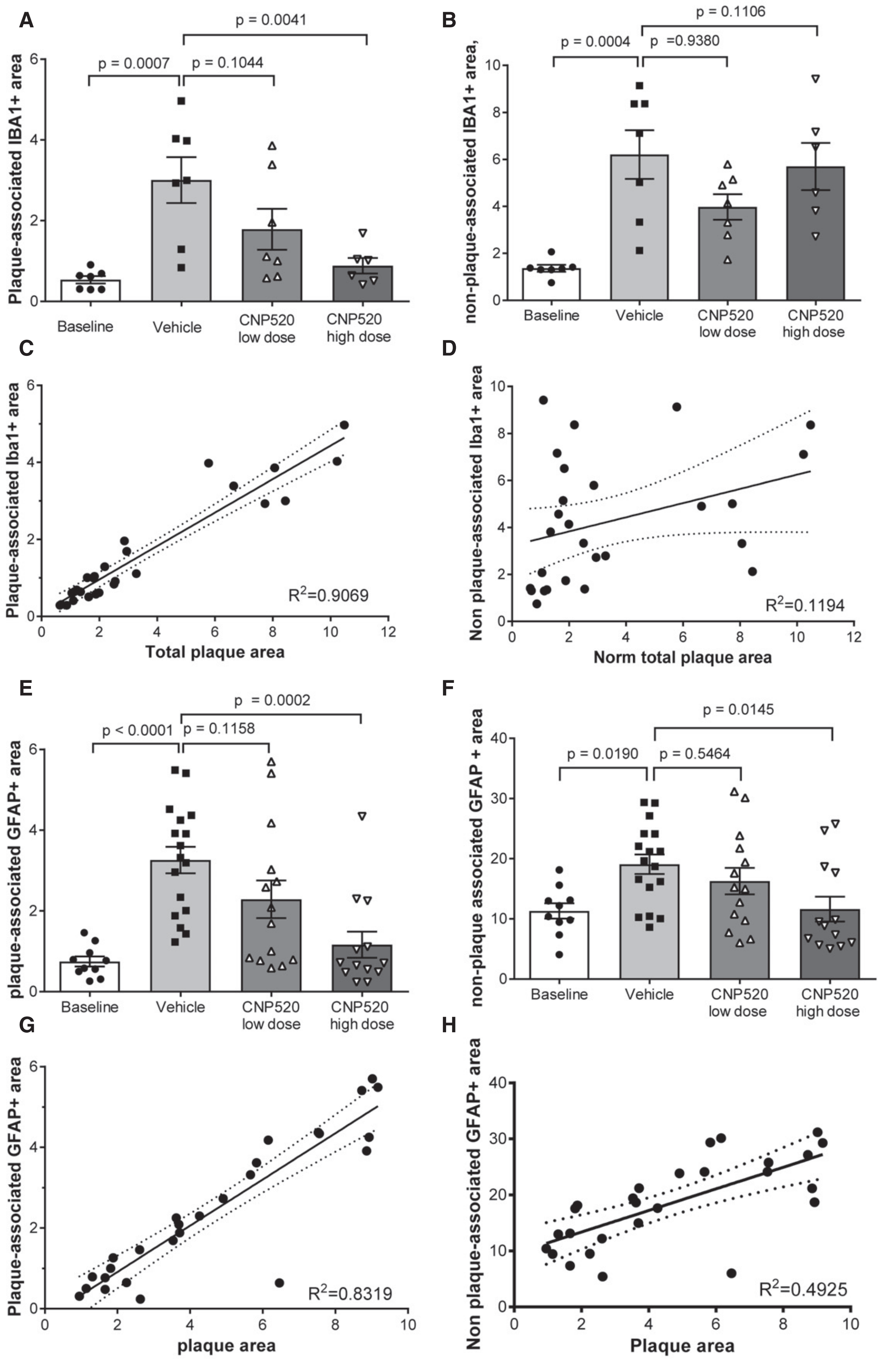
A
p = 0.0041
p = 0.0007
p = 0.1044
Plaque-associated IBA1+ area
Baseline
Vehicle
CNP520 low dose
CNP520 high dose
B
p = 0.1106
p = 0.0004
p =0.9380
non-plaque-associated IBA1+ area,
Baseline
Vehicle
CNP520 low dose
CNP520 high dose
C
Plaque-associated Iba1+ area
R2=0.9069
Total plaque area
D
Non plaque-associated Iba1+ area
R2=0.1194
Norm total plaque area
E
p = 0.0002
p < 0.0001
p = 0.1158
plaque-associated GFAP+ area
Baseline
Vehicle
CNP520 low dose
CNP520 high dose
F
p = 0.0145
p = 0.0190
p = 0.5464
non-plaque associated GFAP + area
Baseline
Vehicle
CNP520 low dose
CNP520 high dose
G
Plaque-associated GFAP+ area
R2=0.8319
plaque area
H
Non plaque-associated GFAP+ area
R2=0.4925
Plaque area

Figure EV2.

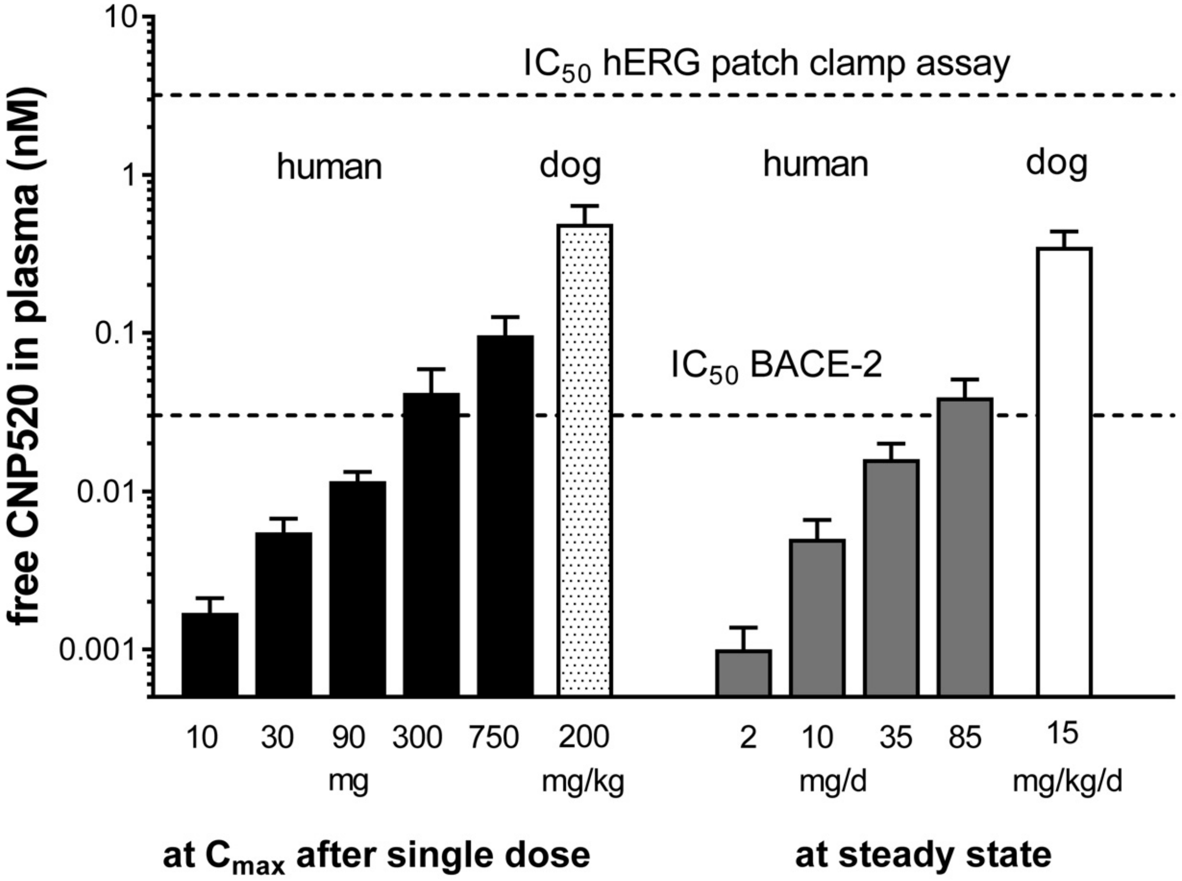

Figure EV3. Free (unbound) CNP520 plasma exposure in humans, relative to the *in vitro* IC_{50} value for hERG and BACE-2 inhibition.

Shown are maximum free CNP520 levels after a single dose, in humans and in dogs, and steady-state exposure after 3-month dosing in human and 9-month dosing in dogs. Shown is mean ± coefficient of variation. Human single dose: n = 8/dose, human steady state: n = 25/dose, dog single dose: n = 4, dog steady state: n = 5.

 © 2018 Novartis AG
